# SmartWand: Validating a Novel Device for Remote Pelvic Floor Physical Therapy

**DOI:** 10.21203/rs.3.rs-9610799/v1

**Published:** 2026-06-05

**Authors:** Aneri Kothari, Jose Bohorquez, Devin Hubbard, Nicole Wiley, Erin Carey

**Affiliations:** University of North Carolina at Chapel Hill; Bold Type Consulting; North Carolina State University; NC TraCS Institute; University of North Carolina Health Care

## Abstract

High-tone pelvic floor dysfunction (HTPFD) affects a substantial proportion of women with chronic pelvic pain and is characterized by hypertonic musculature, myofascial tenderness, dyspareunia, and urinary and defecatory symptoms. Although pelvic floor physical therapy (PFPT) is the first-line treatment, access is severely limited: only 2.47% of U.S. zip codes have even one trained pelvic floor physical therapist, and completion rates at some referral centers are as low as 20%. Existing telehealth and home biofeedback tools support muscle-strengthening exercises but cannot guide patients to therapist-defined myofascial trigger points or objectively verify that therapeutic depth, rotational orientation, and force are reproduced during home-based treatment—capabilities essential for HTPFD management.

We developed the SmartWand, a Bluetooth-enabled intravaginal device that quantifies insertion depth (cm), rotational orientation (degrees), and applied force (kg/cm^2^) and pairs with a smartphone application to deliver real-time, multimodal guidance. Using a two-phase workflow, a pelvic floor physical therapist first programs patient-specific trigger-point coordinates during an in-clinic session; the patient then independently reproduces those coordinates at home with on-screen directional feedback, auditory confirmation, and haptic cues.

In this prospective validation study, 20 adult women—10 with HTPFD and 10 healthy controls—underwent therapist-guided pelvic floor mapping at eight anatomical sites followed by blinded, independent coordinate reproduction. Inter-rater reliability between therapist-programmed and participant-reproduced measurements was assessed using intraclass correlation coefficients (ICC) from two-way mixed-effects models with subject-specific random intercepts to account for repeated within-subject measures. The SmartWand demonstrated excellent agreement for insertion depth (ICC 0.98; 95% CI 0.971–0.986) and rotational orientation (ICC 0.999; 95% CI 0.998–0.999), and good agreement for applied force (ICC 0.877; 95% CI 0.82–0.916). All ICC values surpassed the prespecified success threshold of 0.65. Critically, reliability was consistent across both participant groups, indicating that the hypertonic musculature and myofascial tenderness characteristic of HTPFD did not impair measurement reproducibility.

These findings establish proof-of-concept that patients can independently perform technique-specific internal myofascial release—previously considered feasible only under direct therapist supervision—with objective, quantitative verification of accuracy. The SmartWand system represents a meaningful technological advance over existing home biofeedback platforms by enabling therapist-programmed spatial targeting and calibrated force delivery at patient-specific trigger points. By digitizing treatment parameters and generating objective adherence data, this technology has the potential to expand guideline-concordant PFPT to the millions of women with HTPFD who currently cannot access or complete in-person care, and to support standardized dosing and longitudinal monitoring in future hybrid telehealth care models. Randomized controlled trials comparing SmartWand-augmented hybrid PFPT to standard in-person therapy are warranted to establish clinical efficacy.

## Introduction

Chronic pelvic pain affects up to 25% of women globally, with high-tone pelvic floor dysfunction (HTPFD) contributing to approximately 80% of these presentations^1,2^. HTPFD is characterized by increased resting tone, impaired relaxation, and myofascial tenderness of the pelvic floor musculature, resulting in dyspareunia, urinary dysfunction, defecatory symptoms, and significantly reduced quality of life^3,4^. Despite its high prevalence and morbidity, access to effective, targeted treatment for HTPFD remains severely limited^4,5^.

Consensus guidelines identify pelvic floor physical therapy (PFPT) as the first-line intervention for HTPFD, typically involving internal myofascial release, neuromuscular re-education, and guided relaxation techniques delivered by specially trained therapists^2,6^. Clinical outcomes demonstrate meaningful improvements in pain, sexual function, and urinary and bowel symptoms^2^. However, access barriers are substantial. A national analysis revealed that only 2.47% of U.S. zip codes have at least one pelvic floor physical therapist^9^. Geographic disparities, cost, insurance coverage limitations, time constraints, and discomfort with internal manipulation further restrict access^7,8^. Even among patients who initiate PFPT for HTPFD, adherence to the full protocol remains suboptimal, with completion rates as low as 20% in some referral centers^10^.

Home-based pelvic floor therapy programs are commonly prescribed to supplement in-clinic treatment, yet adherence to unsupervised regimens is often inadequate^11^. Patients frequently report uncertainty about whether they are performing exercises correctly, particularly when internal muscle coordination and force modulation are required. Existing home biofeedback tools such as surface electromyography (sEMG) and pressure perineometry provide feedback on pelvic floor muscle contraction^12^, but lack critical functionality: they cannot guide patients to therapist-defined myofascial trigger points, nor can they objectively verify that patients reproduce the precise spatial coordinates and calibrated force prescribed by their therapists. Effective HTPFD therapy requires precise internal positioning, controlled depth of insertion, rotational orientation, and graded force application during myofascial release^2,13^-capabilities no validated home-based system provides.

Telehealth-delivered PFPT has emerged as one approach to addressing access barriers. Systematic reviews demonstrate that telehealth interventions for pelvic floor dysfunction improve urinary symptoms, pelvic floor muscle function, and quality of life, with efficacy comparable to in-person care^14^. However, the majority of telehealth PFPT research focuses on pelvic floor muscle strengthening exercises for urinary incontinence rather than therapist-directed internal myofascial techniques required for HTPFD management^14^. This represents a critical gap where the patients with the greatest barriers to in-person care–those requiring internal, trigger point–specific therapy–have the fewest validated home-based options.

To address this unmet need, we developed the SmartWand system, an interactive intravaginal device integrated with a Bluetooth-connected mobile application. The system allows physical therapists to program individualized treatment coordinates and force targets corresponding to patient-specific myofascial trigger points. During home-based sessions, patients receive real-time visual feedback, guiding reproduction of the therapist-defined coordinates and force application, enabling independent execution of therapist-prescribed internal myofascial release techniques. The objective of this study was to establish the inter-rater reliability of the SmartWand system by comparing therapist-programmed treatment parameters with patient-reproduced measurements of insertion depth, rotational orientation, and applied force in women with and without HTPFD.

## Methods

### Study Design

This prospective validation study was conducted between May 17th, 2024 and July 20th, 2024 at a private research facility in Orlando, FL. The study evaluated inter-rater reliability between programmed and patient-reported pelvic floor therapy parameters using the SmartWand system. The study protocol was approved by a commercial Institutional Review Board, Sterling IRB (IRB # 10872), relied on by the University of North Carolina (UNC) and all participants provided written informed consent.

### Participants

We enrolled 20 adult women using convenience sampling in a 1:1 case-control design: 10 with HTPFD (cases) and 10 healthy controls. Cases were recruited through IRB-approved social media ads. Cases were eligible if they were English-literate women aged ≥ 18 years with HTPFD diagnosed by a pelvic floor physical therapist, defined as myofascial point tenderness on internal examination with associated pain symptoms (dyspareunia, pelvic pain, urinary dysfunction, or defecatory symptoms). Controls were eligible if they were English-literate women aged ≥ 18 years without a diagnosis of HTPFD, chronic pelvic pain, or dyspareunia, or current pelvic floor physical therapy for any indication.

Exclusion criteria for all participants included current pregnancy or less than 12 months postpartum, active, untreated vulvar dermatologic conditions or vaginal infections, untreated genitourinary syndrome of menopause, atrophic vaginitis, or vulvovaginal atrophy. Participants with treatable conditions could undergo treatment and be re-evaluated for enrollment upon symptom resolution.

### SmartWand System Description

The SmartWand is a Bluetooth-enabled intravaginal device with integrated sensors that quantify three spatial and force parameters during pelvic floor therapy: insertion depth (measured in centimeters), rotational orientation (measured in degrees along xy/yz axes), and applied force (measured in kg/cm^2^).

The device was developed under ISO13485 certified quality management system standards and is constructed using an outer layer made from medical-grade silicone (Elkem LSR-4340) with a biocompatibility profile appropriate for intravaginal use.

The SmartWand connects wirelessly to a proprietary application that supports a two-phase workflow. Its intended use is as follows: During an in-clinic session, the pelvic floor physical therapist performs a standardized internal examination to identify patient-specific myofascial trigger points. Using the SmartWand, the therapist then programs target coordinates for each identified trigger point location, recording the precise depth, rotational orientation and therapeutic force required for effective myofascial release at that site. These parameters are saved within the patient’s application profile. During subsequent home-based sessions, the patient will insert the SmartWand and navigate it to the programmed trigger point locations using real-time visual feedback displayed on the smartphone screen. The application will provide dynamic graphical guidance showing the patient’s current position relative to the therapist-defined target coordinates. When the patient successfully reaches and maintains the target depth, rotation, and force within pre-specified tolerance ranges, the system will deliver multimodal confirmation through audible cues (a “ding” tone), haptic vibration, and visual indicators on the screen. This closed-loop feedback system will enable patients to independently reproduce therapist-prescribed internal myofascial release techniques with objective verification of technique accuracy. For this study, an equivalent version of the app was modified for clinical research and installed on a small laptop computer.

### Study Procedures

Each participant completed a single 60-minute study visit consisting of baseline assessments, therapist-guided pelvic floor mapping, independent patient reproduction of mapped coordinates and postprocedure evaluation. After providing written informed consent, participants completed a demographic survey and medical history questionnaire. Women of childbearing potential underwent pregnancy testing prior to device use.

#### Phase 1: Therapist-guided pelvic floor mapping.

A trained, certified pelvic floor physical therapist performed standardized pelvic floor mapping using the SmartWand at eight anatomical sites: pubococcygeus, iliococcygeus, and obturator internus (bilaterally), plus anterior vaginal wall (12 o'clock position overlying the bladder) and posterior vaginal wall (6 o’clock position overlying the rectum). These sites were selected to represent a broad spectrum of pelvic floor musculature commonly involved in HTPFD and two sites patients are instructed to avoid- bladder and rectum.

For each anatomical site, the physical therapist followed a standardized five-step protocol: (1) identification of the target muscle in the application; (2) device calibration by pressing the calibration button to zero baseline measurements at the vaginal entrance(3) SmartWand insertion to the target depth position (4) adjusted rotational angle to achieve the optimal orientation to each specific muscle; and (5) applied three sequential force thresholds (1, 2, and 3 kg/cm^2^) to assess participant force tolerance. Following force titration, the therapist programmed a final target force for each site that produced palpable tissue engagement and participant-reported pressure sensation without pain. This individualized force threshold was recorded as the therapeutic target for that site. A standardized 1-minute rest interval separated measurements between sites to minimize patient fatigue and discomfort.

#### Phase 2: Patient-guided coordinate reproduction.

Following completion of therapist mapping, participants independently reproduced all eight programmed coordinates using only real-time visual feedback from the smartphone application, without verbal or physical guidance from the therapist. Participants were blinded to the specific numeric values of target coordinates (depth, rotation, and force) and instead relied on the application's graphical interface showing directional arrows and proximity indicators.

For each site, participants: (1) inserted the SmartWand; (2) navigated to the target depth and rotational orientation using on-screen guidance; (3) applied force until reaching the programmed threshold; and (4) maintained the target position and force for 10 seconds to allow measurement stabilization. The application automatically recorded depth, rotation, and force values during this 10-second hold period for subsequent comparison with therapist-programmed reference values. The order of site reproduction matched the therapist mapping sequence to maintain consistency.

#### Post-Procedure Assessment.

Upon completion of both phases, participants completed an electronic acceptability questionnaire assessing comfort, tolerability, ease of use, and willingness to use the SmartWand system for home-based therapy.

### Outcome Measures

The primary outcome measured was the inter-rater reliability between therapist-programming and participant-reproduced measurements, quantified using intraclass correlation coefficients (ICC) for three parameters: insertion depth (cm), rotational orientation (degrees), and applied force (kg/cm^2^). ICCs were calculated for each parameter both overall and stratified by participant group (HTPFD vs. healthy controls). Secondary outcomes included the relationship between measurement parameters (insertion depth, orientation) and participant-reported pain or discomfort during reproduction. Device usability and acceptability were also assessed through post-session questionnaires evaluating comfort, ease of use, confidence in reproducing therapist-prescribed positions, and willingness to use the system for home-based therapy.

### Statistical Analysis

Sample size was calculated to detect clinically meaningful reliability with adequate precision. Based on prior validation studies of biofeedback devices for pelvic floor therapy, we hypothesized an ICC of at least 0.80 for each measurement parameter. Power analysis determined that 20 participants (10 per group) would yield a 90% confidence interval width of at most ± 0.15 around observed ICC values ≥ 0.80, providing sufficient precision to distinguish good from excellent reliability.

ICCs were calculated using two-way mixed effects models with agreement to assess concordance between therapist-programmed and patient-reproduced measurements. To account for the clustered data of eight anatomical sites per participant with bilateral measurements for six of eight sites, models, included subject-specific random intercepts. This approach appropriately handles within-subject correlation arising from repeated measurements. ICC were calculated separated for insertion depth, rotational orientation (xy-plane and yz-plane analyzed independently), and applied force. ICC values were interpreted using established thresholds: <0.50 = poor agreement; 0.50–0.75 = moderate; 0.75–0.90 = good; >0.90 = excellent. The predefined success criterion was an ICC ≥ 0.65 with 90% confidence for each measured parameter, indicating at least moderate reliability with precision sufficient to rule out poor agreement. Usability and acceptability data from post-session questionnaires were summarized using descriptive statistics. Statistical analyses were performed using **R**.

## Results

### Participant Characteristics

Twenty women were enrolled and completed the study: 10 with HTPFD and 10 healthy controls. Mean age of healthy controls was 39.1 (SD 13.1) and 38.3 (SD 7.2) in the HTPFD group. Both cohorts were racially and ethnically diverse. Hispanic white participants were the most represented group in both arms (30% controls, 40% HTPFD), followed by Black/African American participants in controls (30%) and mixed/multiracial participants in the HTPFD group (20%). Non-Hispanic white participants comprised 20% of controls and 10% of the HTPFD group. No Asian participants were enrolled in the control arm, while one (10%) was included in the HTPFD group. One participant in each group preferred not to disclose race/ethnicity. (Table 1).

### Inter-Rater Reliability

**Figure 1:**
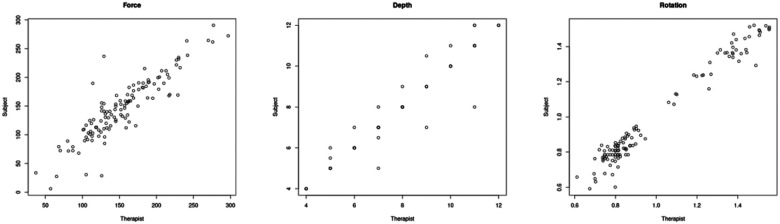
Scatter plots of therapist-programmed versus patient-reproduced values for applied force (left), insertion depth (center), and rotational orientation (right). Each point represents one measurement. Points clustering near the identity line indicate high concordance between therapist-programmed targets and patient-reproduced values across all three parameters.

**Figure 2:**
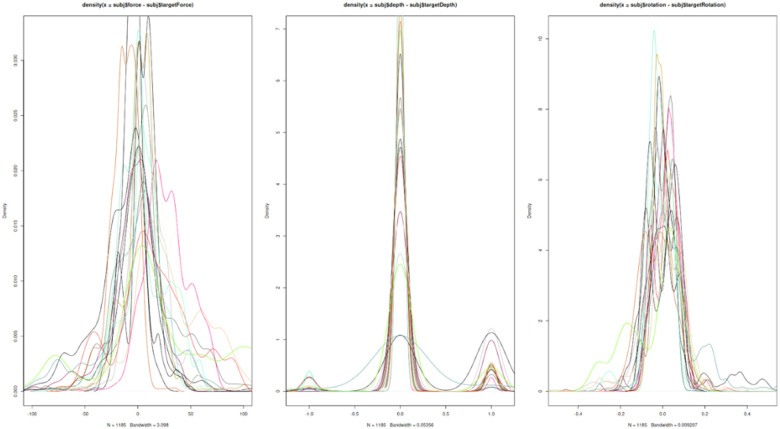
Kernel density plots (specifically per-subject error distribution curves) showing the distribution of the difference between patient-reproduced and therapist-programmed values for applied force (left), insertion depth (center), and rotational orientation (right), shown separately for each participant. The narrower distributions for depth and rotation reflect their excellent intraclass correlation coefficients.

The SmartWand system demonstrated strong inter-rater reliability between therapist-programmed and patient-reproduced measurements across all three parameters (Table 2). For insertion depth, the ICC was 0.98 (95% CI: 0.971–0.986), indicating excellent agreement. For rotational orientation, the ICC was 0.999 (95% CI: 0.998–0.999), also indicating excellent agreement. For applied force, the ICC was 0.877 (95% CI: 0.82–0.916), indicating good agreement. All ICC values exceeded the predefined success criterion of 0.65 with 90% confidence. Scatter plots of therapist-programmed versus patient-reproduced values confirmed high concordance across all three parameters, with data points clustering near the identity line in each case (Fig. 1). Kernel density plots revealed excellent intraclass correlation coefficients for depth and rotation, reflected in the narrow distribution of the difference between patient-reproduced and therapist-programmed values (Fig. 2).

### Comparison Between HTPFD and Healthy Controls

Inter-rater reliability was consistent across participant groups. ICC values for applied force, insertion depth, and rotational orientation did not differ meaningfully between women with HTPFD and healthy controls. This indicates that pelvic floor pathology including the hypertonic musculature and myofascial tenderness that is characteristic of HTPFD did not impair participants’ ability to accurately reproduce therapist-programmed coordinates or force targets using the SmartWand guidance interface.

## Discussion

### Principal Findings

This prospective validation study demonstrated that patients can accurately reproduce therapist-programmed pelvic floor therapy parameters using the SmartWand system with strong inter-rater reliability across all measured domains. Intraclass correlation coefficients exceeded predefined success thresholds of 0.65, with excellent agreement for depth (ICC 0.938, 90% CI: 0.916–0.950) and rotational orientation (ICC 0.991, 90% CI: 0.988–0.993), and good agreement for force (ICC 0.841, 90% CI: 0.788–0.869). Critically, performance was consistent in both women with HTPFD and healthy controls, indicating that symptom status did not impair measurement reproducibility.

### Clinical Implications

Pelvic floor physical therapy is the evidence-based first-line treatment for HTPFD, yet access barriers are severe and worsening, Only 2.47% of U.S. zip codes have even one pelvic floor physical therapist, and among patients who successfully initiate PFPT, completion rates as low as 19% in some referral centers^9,10^. Geographic distance, cost, time constraints, insurance limitations, and patient discomfort with in-person internal manipulation create compounding barriers that leave the majority of affected women without access to guideline-concordant care. This study provides foundational evidence that patients can independently perform therapist-prescribed internal myofascial release using objective, quantitative feedback–a capability previously assumed to require direct therapist supervision.

For HTPFD, therapeutic benefit depends on precise localization of hypertonic musculature/trigger points and application of graded, sustained pressure to achieve myofascial release^13^. Without objective feedback, patients performing home exercise may apply insufficient force (limiting efficacy), excessive force (causing pain and discontinuation or target incorrect anatomical locations entirely. The SmartWand system addresses this fundamental limitation by providing real-time verification that patients have achieved the correct depth, rotational angle, and therapeutic force at each programmed trigger point.

### Expanding the Scope of Telehealth Pelvic Floor Therapy

Existing evidence supports telehealth-delivered PFPT for certain conditions, particularly pelvic floor muscle strengthening for stress urinary incontinence, where remotely guided Kegel exercises demonstrate non-inferiority to in-person care^14,15^. However, the majority of telehealth PFPT research focuses on voluntary muscle contraction and relaxation exercises performed without internal manipulation^14^. This represents a significant technical and clinical gap where women with HTPFD require internal myofascial release at specific trigger points–a technique that has been considered feasible only with direct therapist palpation and real-time manual guidance.

Current home biofeedback devices, including surface electromyography (sEMG), pressure perineometry, and recent accelerometer-based systems, provide valuable feedback for pelvic floor muscle training and have demonstrated efficacy for strengthening protocols^12,16–18^. However, these technologies detect muscle activation and global motion without the ability to (1) target therapist-programmed anatomical coordinates corresponding to patient-specific trigger points; (2) validate that patients have reproduced the spatial position defined by the therapist; or (3) confirm application of calibrated force thresholds individualized to each muscle site. The SmartWand system represents a distinct technological advancement by enabling therapist-programmed spatial coordinates and calibrated force application that patients can independently reproduce with objective verification. This expands the technical scope of remote PFPT from muscle training to targeted myofascial release—a treatment modality previously deliverable only in person.

### Enabling Standardization and Longitudinal Monitoring

Beyond immediate treatment delivery, the quantitative nature of SmartWand measurements creates opportunities for standardized dosing protocols and objective progress tracking. Manual pelvic floor therapy relies on subjective therapist palpation and patient-reported sensation, which introduces variability across providers and limits ability to measure adherence or technique drift over time. By digitizing treatment parameters–recording when patients performed therapy, which trigger points they targeted, whether they achieved prescribed coordinates, and how long they maintained therapeutic force–the system generates objective data that can inform clinical decision-making, identify patients requiring technique refinement, and provide evidence of therapeutic engagement for insurance documentation.

### Strengths and Limitations

This study has several methodological strengths. The prospective design with predefined success criteria and confidence intervals minimizes post-hoc analytical bias. Inclusion of both symptomatic (HTPFD) and asymptomatic participants with consistent inter-rater reliability across groups demonstrates that the technology performs reliably regardless of pelvic floor pathology. Assessment of three independent measurement parameters—depth, rotation, and force—provides comprehensive validation of the system's spatial and mechanical accuracy. Use of mixed-effects models appropriately accounts for the clustered data structure arising from multiple measurements per participant. Several limitations warrant acknowledgment. The sample size was modest (n = 20) and derived from convenience sampling, which may limit generalizability. All participants were English-literate and able to navigate smartphone applications; accessibility for non-English speakers, older adults less familiar with mobile technology, or individuals with visual or cognitive impairments requires dedicated study. The study evaluated short-term reproducibility within a single session and was not designed to assess longitudinal adherence or clinical outcomes. The study used a prototype device under therapist supervision; real-world implementation may introduce additional variability. Force application showed greater variability than depth and rotation (ICC 0.841 vs 0.938 and 0.991), warranting attention in future training protocols potentially including practice sessions with graduated force targets and haptic feedback refinement to improve proprioceptive learning.

### Future Directions

Building on this validation study, the next step is a multisite, non-inferiority randomized controlled trial comparing hybrid SmartWand-augmented PFPT to standard fully in-person PFPT with a mechanical commercially available wand. We propose a pragmatic design enrolling women with HTPFD randomized to 8 weeks PFPT: (1) standard in-person PFPT (weekly 60-minute sessions); or (2) hybrid SmartWand-augmented therapy (in-person sessions weeks 1,2, 4 and 8 for therapist assessment and coordinate programming, with intervening remote sessions weeks 3, 5, 6 and 7 using the SmartWand). Primary outcomes would include a Visual Analog Scale (VAS) and the Genitourinary Pain Index (GUPI). Secondary outcomes would assess sexual function (Female Sexual Function Index), treatment adherence, patient satisfaction, cost-effectiveness from healthcare system and societal perspectives, and therapist workflow integration. The methodological approach demonstrated here, using inter-rater reliability to validate reproducibility of therapist-programmed parameters, provides a framework for validation studies of similar digital therapeutic technologies. Studies across diverse populations and practice settings are necessary to confirm generalizability.

## Conclusion

The SmartWand system demonstrated strong validity for reproducing therapist-defined depth, rotational orientation, and applied force parameters during internal pelvic floor myofascial release therapy. By enabling patients to independently perform technique-specific therapy with objective verification of accuracy, this technology addresses a fundamental barrier to home-based treatment for HTPFD. These findings establish proof-of-concept for remote delivery of internal myofascial release–a treatment modality previously considered feasible only under direct therapist supervision–and support progression to clinical efficacy trials. If validated in randomized controlled trials, SmartWand-augmented hybrid care models could meaningfully expand access to evidence-based pelvic floor physical therapy for the millions of women affected by HTPFD who currently cannot access or complete treatment.

## Figures and Tables

**Table 1:** Participant Demographics

Age, mean (SD)	Healthy Controls	HTPFD
	(n = 10)	(n = 10)
	39.1 (13.1)	38.3 (7.2)
**Race/ethnicity, n (%)**		
White / Caucasian (non-Hispanic)	2 (20%)	1 (10%)
White / Hispanic	3 (30%)	4 (40%)
Black / African American	3 (30%)	1 (10%)
Mixed / Multiracial	1 (10%)	2 (20%)
Asian	0 (0%)	1 (10%)
Prefers not to say	1 (10%)	1 (10%)

Age not stated for 1 HTPFD participant; mean and SD calculated on n = 9. Mixed/Multiracial includes: Mixed, Biracial, White/Filipino

**Table 2:** Inter-Rater Reliability Between Therapist-Programmed and Patient-Reproduced Measurements

Parameter	ICC	95% Confidence Interval	Interpretation
Applied Force (N)	0.877	0.82–0.916	Good
Insertion Depth (mm)	0.98	0.971–0.986	Excellent
Rotational Orientation (degrees)	0.999	0.998–0.999	Excellent

ICC = intraclass correlation coefficient. Interpretation: <0.50 = poor; 0.50–0.75 = moderate; 0.75–0.90 = good; >0.90 = excellent.
